# Dose-specific efficacy of adipose-derived mesenchymal stem cells in septic mice

**DOI:** 10.1186/s13287-023-03253-3

**Published:** 2023-02-19

**Authors:** Kui Li, Tao Wang, Rui Li, Fulai Xue, Guodan Zeng, Jingyao Zhang, Yuan Ma, Li Feng, Y. James Kang

**Affiliations:** 1 Regenerative Medicine Research Center, Sichuan University West China Hospital, Chengdu, Sichuan 610041 China; 2 Memphis Institute of Regenerative Medicine, University of Tennessee Health Science Center, Memphis, TN 38163 USA; 3 Laboratory of Ethnopharmacology, Tissue-orientated Property of Chinese Medicine Key Laboratory of Sichuan Province, Sichuan University West China Hosipital, Chengdu, Sichuan 610041 China

**Keywords:** Sepsis, Adipose-derived mesenchymal stem cells, Dose-specific effect, Phagocytosis, Coagulation

## Abstract

**Background:**

Mesenchymal stem cells (MSCs) therapy for sepsis has been extensively studied in the past decade; however, the treatment regimen and mechanism of action of MSCs remain elusive. Here, we attempted to understand the efficacy and mechanism of action of MSCs on rescuing mice with sepsis.

**Methods:**

A mouse model of sepsis was produced by cecal ligation and puncture (CLP). Allogeneic adipose-derived MSCs (ADSCs) were administered by intravenous infusion at 6 h after CLP, and dose-related effects of ADSCs on these mice were determined by survival rate, histopathological changes, biochemical and coagulation parameters, bacterial load, and plasma levels of endotoxin and inflammatory cytokines. The tissue distribution of intravenously infused ADSCs in septic mice was investigated by pre-labeling ADSCs with the lipophilic membrane dye PKH26. RNA sequencing analysis was performed to assess the transcriptional changes in peripheral blood mononuclear cells (PBMCs) and the liver.

**Results:**

A significant therapeutic effect of ADSCs at a dose of 2 × 10^7^ cells/kg in septic mice was evidenced by a remarkable reduction in mortality (35.89% vs. 8.89% survival rate), blood bacterial burden, systemic inflammation, and multiple organ damage. In contrast, ADSCs at a lower dose (1 × 10^7^ cells/kg) failed to achieve any beneficial outcomes, while ADSCs at a higher dose (4 × 10^7^ cells/kg) caused more early death within 24 h after CLP, retaining a steady survival rate of 21.42% thereafter. PKH26-labeled ADSCs were predominantly localized in the lungs of septic mice after intravenous infusion, with only a smaller proportion of PKH26-positive signals appearing in the liver and spleen. RNA sequencing analysis identified that insufficient phagocytic activity of PBMCs in addition to a hyperactivation of the hepatic immune response was responsible for the ineffectiveness of low-dose ADSCs therapy, and acute death caused by high-dose ADSCs infusion was associated with impaired coagulation signaling in PBMCs and exacerbated hepatic hypoxic injury.

**Conclusions:**

Our findings demonstrate a dose-specific effect of ADSCs on the treatment of sepsis due to dose-related interactions between exogenous stem cells and the host’s microenvironment. Therefore, a precise dosing regimen is a prerequisite for ADSCs therapy for sepsis.

**Supplementary Information:**

The online version contains supplementary material available at 10.1186/s13287-023-03253-3.

## Background

Sepsis, defined as a life-threatening organ dysfunction condition caused by a dysregulated host response to infection, remains a leading cause of morbidity and mortality worldwide [1]. Sepsis is generally manifested by both sustained hyperinflammation and immune suppression, ultimately evolving to acute organ failure, coagulation disorders, and hypotension [2]. Symptomatic management, including fluid resuscitation, antibiotic administration, and vasopressors, is the mainstay of treatment for sepsis, but a more effective treatment is on demand [3].

Mesenchymal stem cells (MSCs) have recently been recognized as a promising treatment for sepsis due to their antibacterial, immunomodulatory, and organ-protective properties [4]. Over the past decade, numerous studies have reported the therapeutic effect of MSCs on reducing mortality in animal models of sepsis; however, the efficacy of MSCs therapy in these preclinical studies is rather vague due to ambiguous MSCs treatment procedures [5, 6]. In this context, a systematic evaluation of the impact of differential MSCs treatment procedures on sepsis, including the route, timing, and dose of MSCs administration, becomes a prerequisite for clinical translation.

A consensus has been gradually building up on the optimal timing and route of MSCs administration in experimental sepsis, i.e., intravenous (IV) infusion within 6 h after sepsis induction [5, 6]. However, the optimal dosing regimen of MSCs for the treatment of sepsis remains controversial.

A recent systematic review of clinical trials reporting positive outcomes for the treatment of multiple diseases with IV MSCs indicated that the minimum effective dose (MED) of MSCs ranged from 100 to 150 million cells/patient (equal to 1.4 × 10^6^ to 2.1 × 10^6^ cells/kg), with lower and higher doses being less effective [7]. This raises a new perspective that IV MSCs may exert dose-specific efficacy in improving clinical outcomes in human disease. In addition, several recently published phase I trials showed that IV administration of MSCs up to a dose of 3 × 10^6^ cells/kg was safe and well tolerated in patients with severe sepsis but failed to improve mortality and systemic inflammatory biomarkers [8–11]. Therefore, conducting a systematic dose–response study to determine the MED range of MSCs in the treatment of sepsis will provide an important reference for optimizing the clinical dosing regimen of MSCs.

In the present study, we employed cecal ligation and puncture (CLP)-induced polymicrobial sepsis in mice, which is considered the gold standard animal model for studying clinical sepsis [12]. Adipose-derived MSCs (ADSCs), one of the most widely used sources of MSCs, were used for the treatment of experimental sepsis. Given that the effective IV dose of MSCs in septic mice reported in preclinical studies ranged from 2.5 × 10^5^ to 1 × 10^6^ [5, 6], this corresponds to a range of 1 × 10^7^ to 4 × 10^7^ cells/kg body weight for a 25 g mouse. We thus chose three different IV doses, including low-dose (1 × 10^7^ cells/kg), medium-dose (2 × 10^7^ cells/kg), and high-dose (4 × 10^7^ cells/kg), to investigate the dose-response effect of allogeneic ADSCs in CLP-induced septic mice. We hypothesized that ADSCs might exert a dose-specific effect on sepsis-induced mortality, systemic inflammation, and multi-organ damage. The dynamic distribution of intravenously infused ADSCs in septic mice was assessed by pre-labeling ADSCs with the lipophilic membrane dye PKH26. Transcriptomic analysis of peripheral blood mononuclear cells (PBMCs) and liver was performed to unravel the molecular basis underlying the dose–response effect of ADSCs.

## Materials and methods

### Animals

Male C57BL/6J mice were obtained from Dashuo Biological Technology Co., Ltd. (Chengdu, China) and housed at the Experimental Animal Center of West China Hospital on a 12 h light–dark cycle with ad libitum access to food and water. All animal procedures were approved by the Animal Care Committee at Sichuan University West China Hospital (protocol No. 20211135A), following the Guidelines for the Care and Use of Laboratory Animals of the National Institutes of Health.

### Sepsis induction and characterization

A mouse model of sepsis was induced by cecal ligation and puncture (CLP) as described in previous studies [13], with some modifications to generate a more clinically relevant severe sepsis. Briefly, male C57BL/6J mice (weight 25–30 g, age 8–12 weeks) were anesthetized with 3% isoflurane by inhalation induction and maintained on 1.5% isoflurane during surgery. An incision was made along the midline of the abdomen to expose the cecum. The cecum was ligated with 5–0 silk for a length of 1.0 cm, punctured twice with an 18–gauge needle, and gently squeezed out an appropriate amount of cecum contents to ensure the permeability of the perforation. The cecum was then placed back into the abdominal cavity, and the muscle and skin were sutured with 5–0 silk. Postoperative care, such as fluid resuscitation, was performed by subcutaneous injection of 1 mL of prewarmed saline (37 °C) immediately after the CLP procedure to minimize dehydration and prevent animal shock. Sham-operated mice underwent the same surgical procedure except for ligation and puncture. In addition, sepsis-inducing procedures were performed in the morning of the day to minimize the effect of circadian variation on the results [14, 15].

Early septic symptoms occurred over a 6 h period after CLP induction as determined by core body temperature (Additional file 1: Fig. S1A) and the Murine Sepsis Score (MSS) (Additional file 1: Fig. S1B) [16]. The plasma levels of endotoxin, inflammation-associated cytokines (IL-1*β*, IL-6, IL-10, and TNF-*α*), and biochemical indicators (AST, ALT, LDH, and BUN) were all dramatically elevated in the CLP group compared with the sham group (Additional file 1: Fig. S1C-E). Histological examination showed obvious pathological changes, including tissue destruction, necrosis, and leukocyte infiltration, in the lung, liver and kidney of septic mice (Additional file 1: Fig. S1F). Due to systemic inflammation and multiple organ damage, CLP mice began to die at 12 h after surgery, reaching 60% mortality within 24 h and 90% within 168 h, whereas no mortality was observed in the sham-operated group (Additional file 1: Fig. S1G).

### Mouse ADSCs isolation, characterization and treatment

Male C57BL/6J mice (4 weeks old) were euthanized by cervical dislocation after isoflurane inhalation anesthesia and used as ADSCs donors. Mouse ADSCs were isolated from the inguinal fat pad under sterile conditions as described previously [17]. ADSCs were cryopreserved between passages 2 and 3 for subsequent characterization and therapeutic studies. Cryopreserved ADSCs were thawed, washed, and resuspended in 200 µL of vehicle solution (1% albumin) for intravenous infusion.

The ability of ADSCs to differentiate into adipocytes, osteocytes, and chondrocytes was assessed using cell differentiation kits (Minneapolis, MN, USA) according to the manufacturer’s instructions. Immunophenotyping of ADSCs was performed by flow cytometry analysis after staining with the monoclonal antibodies PE-CD29, FITC-CD44, APC-CD31, and APC-CD45 (BD, USA). In addition, immunofluorescence staining was performed for octamer-binding transcription factor 4 (Oct4) (ab27985, Abcam, UK) to assess the stemness of cultured ADSCs.

Different doses of ADSCs (1 × 10^7^, 2 × 10^7^ or 4 × 10^7^ cells/kg, resuspended in 0.2 mL of 1% albumin solution) or vehicle (1% albumin) were administered intravenously at 6 h after sham or CLP surgery via the tail vein.

### Experimental design

Male C57BL/6J mice were weight-matched (25–30 g) and randomly assigned to each experimental group. Randomization were performed after the surgical procedure (CLP or sham operation) using the RAND() command in Microsoft Excel 2013 software. Briefly, a specific random number between 0 and 1 was generated for each mouse in Sham or CLP group, respectively. Random numbers were then sorted from small to large and assigned to different treatment subgroups in turn.

Specifically, for survival analysis, 16 sham-operated or CLP mice were randomly assigned to four treatment subgroups (Vehicle, ADSCs1, ADSCs2, and ADSCs4) of 4 mice each. Furthermore, to ensure the robustness and reliability of the dose-response effect of ADSCs in improving survival in CLP mice, four additional independent treatment experiments were performed in CLP mice. Survival was monitored every 12 h for 7 days. In addition, mice were preemptively euthanized for humane reasons if they exhibited any of the following signs: emaciation, gasping, unresponsiveness to touch, or anal temperature < 25 °C.

For studies tracking the tissue distribution of intravenously infused ADSCs, 48 CLP mice were randomly assigned to four treatment groups as described above, 12 mice in each group. Mice were killed to harvest organs at 12, 24, and 72 h after CLP surgery (*n* = 3/time point).

For other functional and mechanistic studies, a total of 164 CLP mice were used and randomly assigned to four treatment groups as described above, 41 mice in each group. They were tested biochemical parameters (*n* = 5/group), coagulation function (*n* = 7/group), histopathology (*n* = 5/group), blood bacterial load (*n* = 5/group), plasma levels of endotoxin (*n* = 5/group) and inflammatory cytokines (*n* = 5/group) and transcriptomic changes in PBMCs and liver (*n* = 5/group for RNA-seq analysis and *n* = 4/group for qPCR analysis). In addition, a total of 8 sham-operated mice were used to determine reference baseline values for coagulation parameters (*n* = 5) and plasma inflammatory factor levels (*n* = 3).

Animals that died within 6 h after CLP surgery were excluded from subsequent treatment experiments. Blinding was not performed unless specifically mentioned.

### Tracking of PKH26-labeled ADSCs

ADSCs were labeled with PKH26 (Sigma-Aldrich, USA) in accordance with the manufacturer’s instructions. Different doses of labeled ADSCs were resuspended in 200 µL of vehicle solution (1% albumin) and injected intravenously at 6 h after CLP via the tail vein. Mice were euthanized by CO_2_ inhalation following isoflurane inhalation anesthesia at 12, 24, and 72 h after CLP surgery and perfused through the heart with 10 mL of 0.9% saline at a flow rate of 5 mL/min. Lungs, liver, spleen, heart, and kidneys were collected and fixed in 4% paraformaldehyde for at least 24 h. Then, tissue samples were cut into 2- to 3-mm-thick slices. After washing with PBS, tissue sections were shaken in 0.1% Triton X-100 for 1 h at room temperature, followed by nuclear staining with DAPI (D9542, Sigma-Aldrich, USA) for 1 h. Tissue slices were then cleared in the refractive index matching solution (RIMS) [18] for 24 h at 37 °C and protected from light before mounting and imaging.

Images were acquired using a laser confocal microscope (A1, Nikon) coupled with an imaging analysis system (NIS-Elements software, Nikon), with a full view of each tissue section collected under a × 10 ocular lens and × 4 objective lens (measured resolution: 1200 dpi, scar bar: 500 µm), and three to five magnified fields collected through a × 10 ocular lens and × 20 objective lens (measured resolution: 600 dpi, scar bar: 100 µm).

### Plasma biochemistry analysis

Blood was collected by intracardiac puncture and preserved in EDTA-K2 tubes. Plasma samples were harvested by centrifugation at 1600 g for 15 min at 4 °C. Plasma levels of ALT, AST, BUN, and LDH were quantitatively analyzed by using an autoanalyzer (Roche, Switzerland) in accordance with the instructions of the manufacturer.

### Histologic analysis

Lung, liver, and kidney tissues were collected promptly after mice were killed and fixed in 4% paraformaldehyde for 24 h. After fixation, tissues were embedded in paraffin, sectioned, and stained with hematoxylin and eosin (H&E). Lung tissue samples were additionally stained for fibrin using a commercially available MSB stain kit (G2040, Solarbio, CN). Images were taken with a light microscope (80i, Nikon), processed with imaging software (SPOT Software, version 4.6), and at least three 10 × field (for H&E staining) or 20 × field images (for MSB staining) were taken from individual tissues of each mouse.

Pathology scoring and the number of pulmonary thrombi were blindly assessed by two professional pathologists according to a previous study [19].

### Blood bacterial load and endotoxin determination

Whole blood samples were plated on Columbia blood agar plates (CHROMagar, China) with a 100-fold dilution in sterile saline and incubated overnight at 37 °C. Bacterial numbers were analyzed by colony forming units (CFU).

For plasma endotoxin assays, plasma samples were diluted 1:10 with endotoxin-free water and assayed using a ToxinSensor™ Chromogenic LAL Endotoxin Assay Kit (GenScript, China) following the manufacturer’s recommendations. The absorbance at 545 nm was measured. The concentration of plasma endotoxin was expressed as EU/ml.

### Cytokine immunoassay and multiplex array

Plasma levels of pro-inflammatory (IL-1*β*, IL-6, and TNF-*α*) and anti-inflammatory (IL-10) cytokines in sham-operated or CLP mice were measured by enzyme-linked immunosorbent assay (ELISA) according to the manufacturer’s instructions (R&D Systems, American).

The dose-response effect of ADSCs on sepsis-induced systemic inflammation was determined by examining changes in plasma cytokine levels using the Bio-Plex Pro Mouse Cytokine 23-Plex Assay (Bio-Rad, USA) according to the manufacturer’s instructions, containing the following targets: IL-1*α*, IL-1*β*, IL-2, IL-3, IL-4, IL-5, IL-6, IL-9, IL-10, IL-12 (p40), IL-12 (p70), IL-13, IL-17A, eotaxin, G-CSF, GM-CSF, IFN-*γ*, KC, MCP-1, MIP-1*α*, MIP-1*β*, RANTES, and TNF-*α*. Plasma samples from sham-operated mice were used as reference controls. The expression patterns of these cytokines were visualized by heatmap.

### Isolation of PBMCs from whole blood

Blood diluted 1:1 with PBS was carefully layered on top of Histopaque®-1083 (Sigma, USA) and centrifuged at 400 g for 30 min at room temperature. The milky-white cell layer of PBMCs was gently collected and washed twice with PBS (centrifuge at 250 g for 10 min). After the final wash, PBMCs were lysed in TRIzol reagent (Thermo Fisher, USA) and stored at − 80 °C for further RNA sequencing and qPCR analysis.

### RNA sequencing analysis

Total RNA was extracted from PBMCs and liver samples using TRIzol reagent (Thermo Fisher, USA) according to the manufacturer’s instructions. The concentration of RNA was determined by Gene Quant pro 1000 (Thermo Fisher, USA). Total RNA quality was assessed via RNA integrity number (RIN) using a Bioanalyzer 2100 (Agilent, USA). Then, cDNA libraries were constructed, and the final average insert size was 300 ± 50 bp. RNA sequencing was carried out with the PE150 sequencing pattern via the Illumina NovaSeq™ 6000 (Illumina, USA) platform. The raw reads were filtered using Cutadapt software (https://cutadapt.readthedocs.io/en/stable, version: cutadapt 1.9) to remove low-quality and undetermined bases. After obtaining the clean reads, HISAT2 software (version 2.0.4) was used to map to the reference genome (Additional file 2: Table S1).

Differential gene expression analysis was conducted using the R package edgeR (https://bioconductor.org/packages/release/bioc/html/edgeR.html). The dose–response effect of ADSCs treatment on CLP mice was analyzed using the Wald test in three pairwise comparisons (CLP + ADSCs1 vs. CLP + Vehicle, CLP + ADSCs2 vs. CLP + Vehicle and CLP + ADSCs4 vs. CLP + Vehicle), in which differentially expressed genes (DEGs) were selected as those with a false discovery rate (FDR) adjusted *p* value < 0.05 and a log2-fold change (log2FC) > 1 or < -1. Venn diagrams were employed to show the number of unique and overlapping DEGs between different groups. The collection of DEGs between the vehicle-treated and different doses of ADSCs-treated groups in CLP mice was named ADSCs-responsive genes.

Functional annotation of DEGs was performed using Gene Ontology (GO) enrichment analysis by R (version 4.1.0), considering enriched terms as those with an adjusted *p* value threshold of < 0.05 (Additional file 3: Table S2). KEGG pathway analysis and visualization were carried out using the Pathview Web server (https://pathview.uncc.edu/) [20].

To discover coexpressed gene modules in response to different infusion doses of ADSCs in CLP mice, we performed a weighted correlation network analysis (WGCNA) of 1215 ADSCs-responsive genes in PBMCs (Additional file 4: Table S3) using the R package WGCNA (version 1.70). Correlations between module eigengenes and different doses of ADSCs treatment were considered to be significant based on the criteria (correlation coefficients > 0.5 or <  − 0.5 and *p* value < 0.05). Hub genes of each significant module were considered those with gene significance (|GS|) > 0.35 and module membership (|MM|) > 0.8 and visualized by heatmap (Additional file 5: Table S4). GO analysis of hub genes in significantly correlated modules was then performed.

Protein–protein interaction (PPI) network analysis was performed (https://www.networkanalyst.ca) [21] to identify key driver (KD) genes in the significantly correlated modules responsible for the induction or inhibition of the coagulation pathway in PBMCs following high-dose ADSCs treatment, and Cytoscape (version 3.9.0) was used for gene network visualization.

Genes involved in the phagocytosis process (GO: 0006909) and hypoxia response [22] (Additional file 6: Table S5) intersected with ADSCs-responsive genes in PBMCs and liver, respectively, and were visualized by heatmaps.

### RT-qPCR analysis

Total RNA was extracted from the PBMCs and liver using the TRIzol reagent (Invitrogen, USA) following the manufacturer’s instructions. 1 µg of the extracted RNA was reverse transcribed to cDNA using a HiScript® III All-in-one RT SuperMix Kit (Vazyme, CN). Real-time qPCR was performed using ChamQ SYBR Color qPCR Master Mix (Vazyme, CN) and a Thermo Q6 Real-Time System according to the manufacturer’s instructions. Gene expression relative to that of TATA-box binding protein (TBP) was analyzed for each sample using the 2^−ΔΔCt^ method. The primers used are shown in Table 1.

**Table 1:** RT-qPCR primer sequences

Primer	Forward sequence (5' to 3')	Reverse sequence (5' to 3′)
*Marco*	ACAGAGCCGATTTTGACCAAG	CAGCAGTGCAGTACCTGCC
*Lrp1*	ACTATGGATGCCCCTAAAACTTG	GCAATCTCTTTCACCGTCACA
*Nr1h3*	CTCAATGCCTGATGTTTCTCCT	TCCAACCCTATCCCTAAAGCAA
*Tnf*	AGTCCGGGCAGGTCTACTTT	GGTCACTGTCCCAGCATCTT
*Tlr4*	ACAAACGCCGGAACTTTTCG	GTCGGACACACACAACTTAAGC
*Ccl3*	CACCCTCTGTCACCTGCTCAA	TGGCGCTGAGAAGACTTGGT
*F5*	CGCAACTAAGGCAGTTCTATGT	GCTAGATCGTGGCTTTTCTTTCT
*Itgb3*	CCACACGAGGCGTGAACTC	CTTCAGGTTACATCGGGGTGA
*Vegfa*	AGGCTGCTGTAACGATGAAG	TCTCCTATGTGCTGGCTTTG
*Slc2a1*	CAGTTCGGCTATAACACTGGTG	GCCCCCGACAGAGAAGATG
*P4ha2*	CAGGTACTATGATGTGATGTCCG	AAAGGGTCGCCTTGAGAAGTC
*Tbp*	CCTTGTACCCTTCACCAATGAC	ACAGCCAAGATTCACGGTAGA

### Coagulation assay

Whole blood was collected by cardiac puncture and anticoagulated with 3.2% buffered sodium citrate (1:9). Samples were centrifuged at room temperature (1800 g, 10 min) to separate plasma and immediately stored at − 20 °C. Plasma prothrombin time (PT) and activated partial thromboplastin time (aPTT) were measured by an automated coagulation analyzer (CS-2400, Sysmex, Japan).

### Statistical analysis

Data are expressed as the mean ± SEM. GraphPad Prism 9.0 (GraphPad, USA) was used for graphing and statistical analysis. Differences in sepsis-associated symptoms between the sham and CLP groups were assessed by unpaired *t* test. For comparisons among multiple treatment groups in CLP mice, all data were first tested for homogeneity of variance by Bartlett’s test and then subjected to one-way ANOVA followed by Tukey’s multiple comparison post hoc test. Survival curves were analyzed by a log-rank test. *p* < 0.05 was considered statistically significant.

## Results

All ADSCs (passages 2–3) used in this study showed a spindle-shaped morphology, adherence to cell culture flasks, expression of mesenchymal markers and differentiation into adipocytes, osteoblasts, and chondroblasts (Additional file 7: Fig. S2A-C), in accordance with previously described criteria by the International Society for Cellular Therapy [23]. Immunostaining analysis also demonstrated positive expression of Oct4, a core stemness-associated transcription factor [24], in the nucleus of cultured ADSCs (Additional file 7: Fig. S2D).

### Dose-related effects of ADSCs on septic mice

**Fig. 1:**
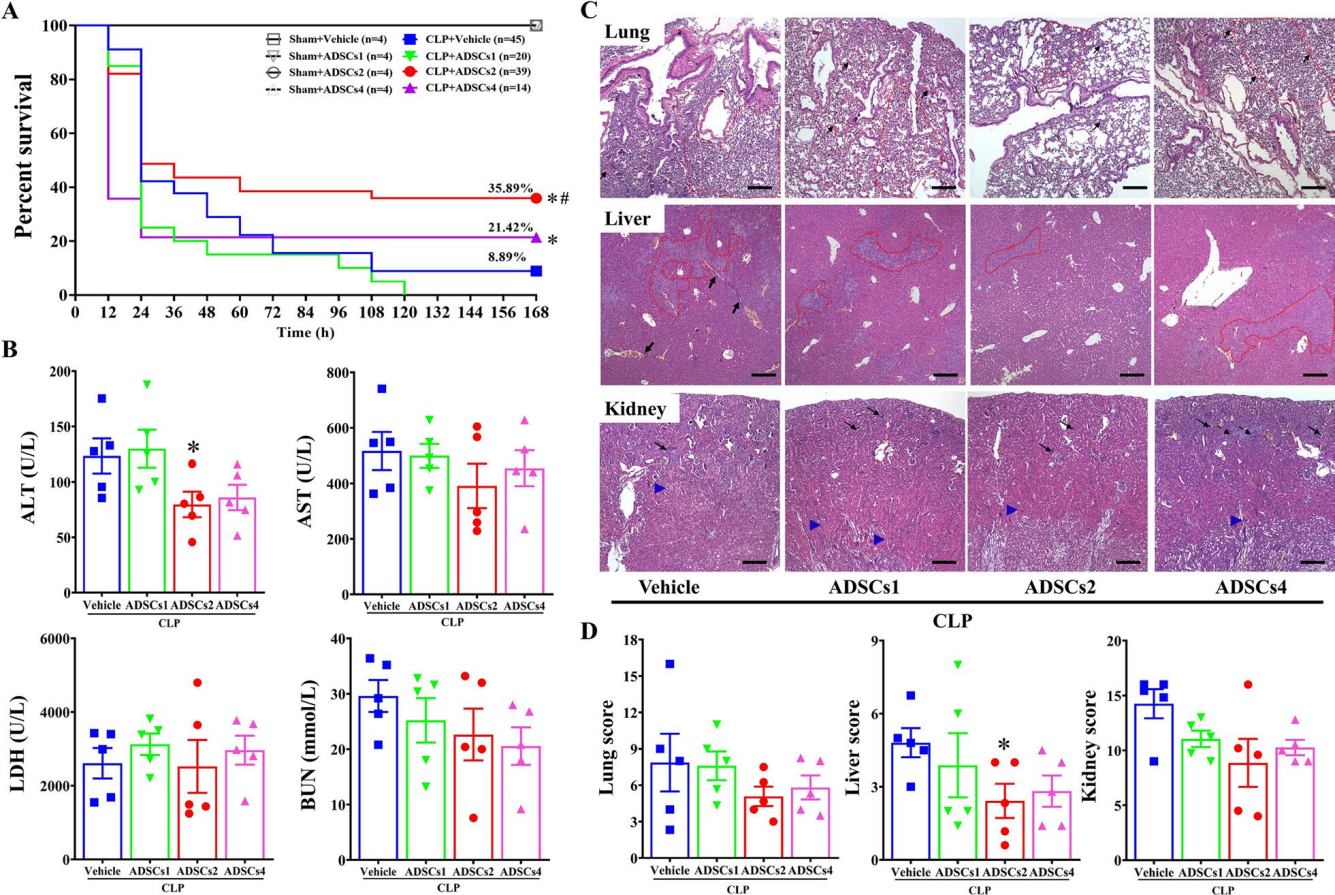
Dose-related effects of ADSCs on CLP-induced mortality and multi-organ damage. Six hours after sham or CLP surgery, mice were intravenously infused with vehicle solution or different doses of ADSCs, including low-dose (1 × 10^7^ cells/kg, ADSCs1), medium-dose (2 × 10^7^ cells/kg, ADSCs2), and high-dose (4 × 10^7^ cells/kg, ADSCs4). The survival rates were monitored every 12 h for 168 h. *n* = 4–45 (**A**). Changes in multi-organ function after ADSCs treatment in septic mice were determined by examining the plasma levels of ALT, AST, BUN, and LDH at 6 h after ADSCs treatment (12 h after CLP). *n* = 5 in each group (**B**). H&E staining was performed to assess the severity of CLP-induced structural damage in multiple organs, including the lung, liver, and kidney, at 6 h after ADSCs treatment. Images were taken by Nikon 80i microscopy and SPOT Software at a resolution of 96 dpi, and processed in Adobe Photoshop at a resolution of 600 dpi. No downstream processing was utilized. Scar bar = 200 μm (**C**). Lung: abnormal alveolar collapse and alveolar septal inflammation (dotted line) and arteriole thrombosis (black arrow); Liver: abnormal hyperplasia of bile duct epithelium (black arrow) and hepatocyte disarrangement and hydropic degeneration (dotted line); Kidney: abnormal acute tubular necrosis (blue triangle, inflammatory infiltration (black dotted arrow) and glomerular damage (black arrow)). Pathology scoring was blindly assessed to indicate the degree of organ damage (**D**). *n* = 5 in each group. *, *p* < 0.05, significantly different from the CLP + Vehicle group; #, *p* < 0.05, significantly different from the CLP + ADSCs4 group

Mice subjected to the CLP procedure started to die at approximately 12 h, and the mortality continued until 108 h after CLP surgery, reaching a steady survival rate of 8.89%. CLP mice receiving allogeneic ADSCs at 2 × 10^7^ cells/kg (ADSCs2) via IV infusion at 6 h after CLP displayed a significant improvement in the survival rate (35.89% vs. 8.89%). However, CLP mice that received ADSCs at 4 × 10^7^ cells/kg (ADSCs4) exhibited more acute death within 24 h after surgery; thereafter, the mortality ceased, retaining a survival rate of 21.42%. In contrast, CLP mice receiving ADSCs at 1 × 10^7^ cells/kg (ADSCs1) displayed continued death beyond 108 h, and all died within 120 h after CLP surgery. There was no mortality in sham-operated control mice receiving any dose of ADSCs (Fig. 1A).

The death peak in CLP-induced septic mice mainly occurred between 12 and 24 h after surgery, probably due to acute multi-organ failure (Additional file 1: Fig. S1G) [25]. Therefore, we examined whether ADSCs had dose-specific protection against multi-organ damage in CLP mice at 6 h after ADSCs treatment (12 h after CLP). As shown in Fig. 1B, the plasma levels of biochemical indicators such as ALT, AST, and BUN tended to decrease in CLP mice treated with ADSCs2 or ADSCs4 compared to the vehicle-treated group, while only the reduction in ALT levels in the ADSCs2 treatment group reached significance. Moreover, histological examination showed that ADSCs2 and ADSCs4 treatment tended to attenuate CLP-induced structural damage in multiple organs, but only ADSCs2 treatment showed a significant amelioration of liver structural abnormalities, including hepatocyte injury and bile duct hyperplasia (Fig. 1C and D). Taken together, these data suggest that intravenously infused ADSCs exhibit dose-specific efficacy in the treatment of sepsis.

### Tissue distribution of intravenously infused ADSCs in septic mice

**Fig. 2:**
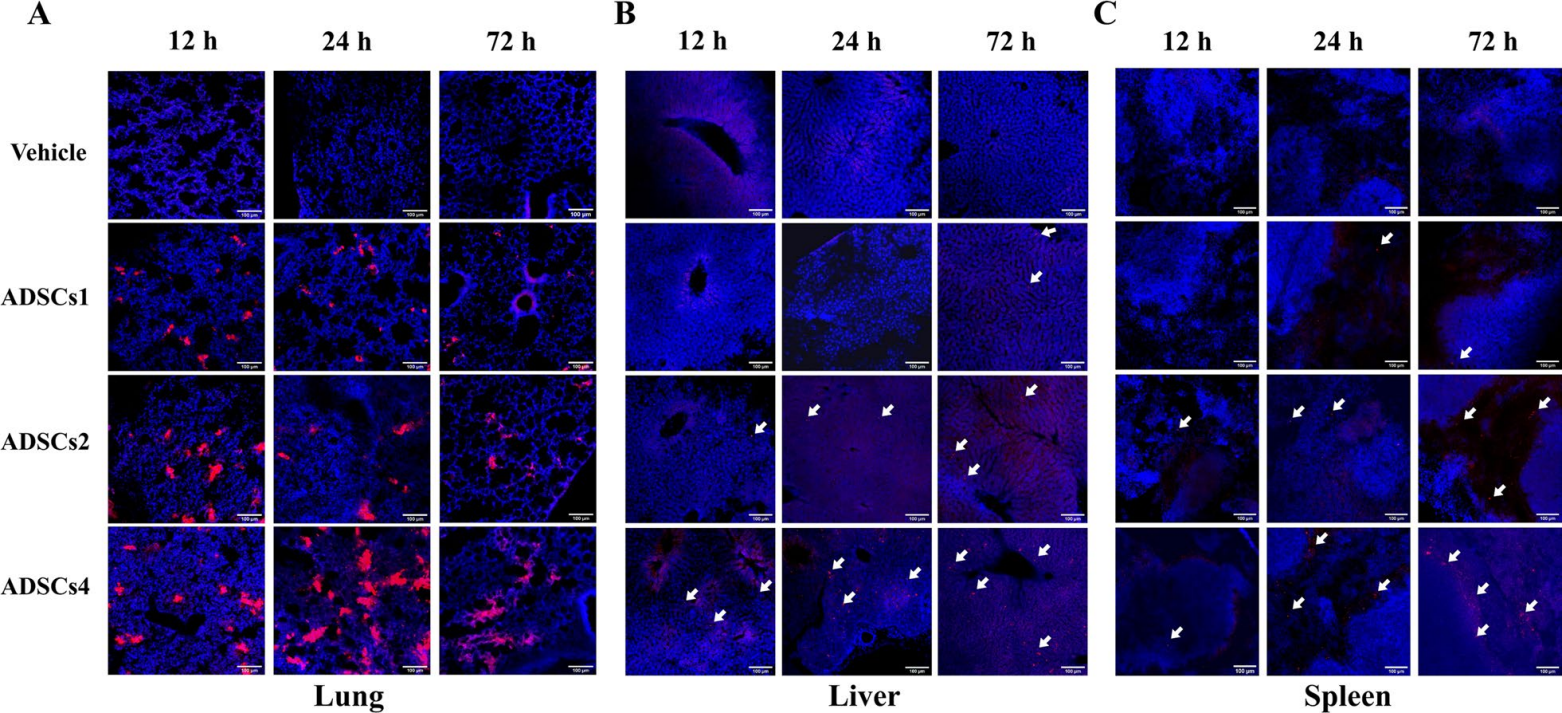
Dynamic distribution of intravenously infused ADSCs in the lung, liver, and spleen of CLP mice. PKH26-labeled ADSCs were injected into CLP mice via the tail vein at 6 h post-CLP to assess their dynamic tissue distribution. PKH26-labeled positive signals (red) were detected in the lung (**A**), liver (**B**), and spleen (**C**) by fluorescence imaging at 12, 24, and 72 h after CLP using a Nikon A1 laser confocal microscope. Images were taken by NIS-Elements software at a resolution of 600 dpi. No downstream processing was utilized. The nucleus was stained with DAPI (blue). CLP mice treated with vehicle solution served as the negative control. *n* = 3 in each group. Scale bar = 100 µm

Considering that the localization and retention of ADSCs in different organs may provide important clues for elucidating the mechanism of the dose-specific effects of ADSCs in septic mice, we labeled ADSCs with PKH26 to assess the tissue distribution of intravenously infused ADSCs at 12, 24, and 72 h after CLP. Six hours after ADSCs infusion (12 h after CLP), the majority of PKH26-positive signals were found in the lungs (Fig. 2A and Additional file 8: Fig. S3A), while smaller proportions were also found in the livers (Fig. 2B and Additional file 8: Fig. S3B) and spleens (Fig. 2C and Additional file 8: Fig. S3C) of CLP mice treated with medium- or high-dose ADSCs. Notably, the amount of PKH26-positive signals in the lung, liver, and spleen all showed a trend of increasing with the infused dose of ADSCs. Moreover, the number of PKH26-positive signals in the lung gradually decreased over time, accompanied by a gradual increase in the liver and spleen. However, no positive signal was detected in the heart (Additional file 8: Fig. S3D) or kidney (Additional file 8: Fig. S3E) throughout the observation period.

### Dose-specific effects of ADSCs on bacterial burden and systemic inflammation in septic mice

Since the intravenously infused ADSCs were predominantly stuck in the lungs of septic mice and only sporadic PKH26-positive signals appeared in the liver at 6 h post-treatment, the protective effect of ADSCs against CLP-induced liver injury may be attributed to their antibacterial and immunomodulatory properties rather than direct involvement in local tissue repair. To test this hypothesis, we examined changes in bacterial burden and systemic inflammation in CLP mice after treatment with different doses of ADSCs.

**Fig. 3:**
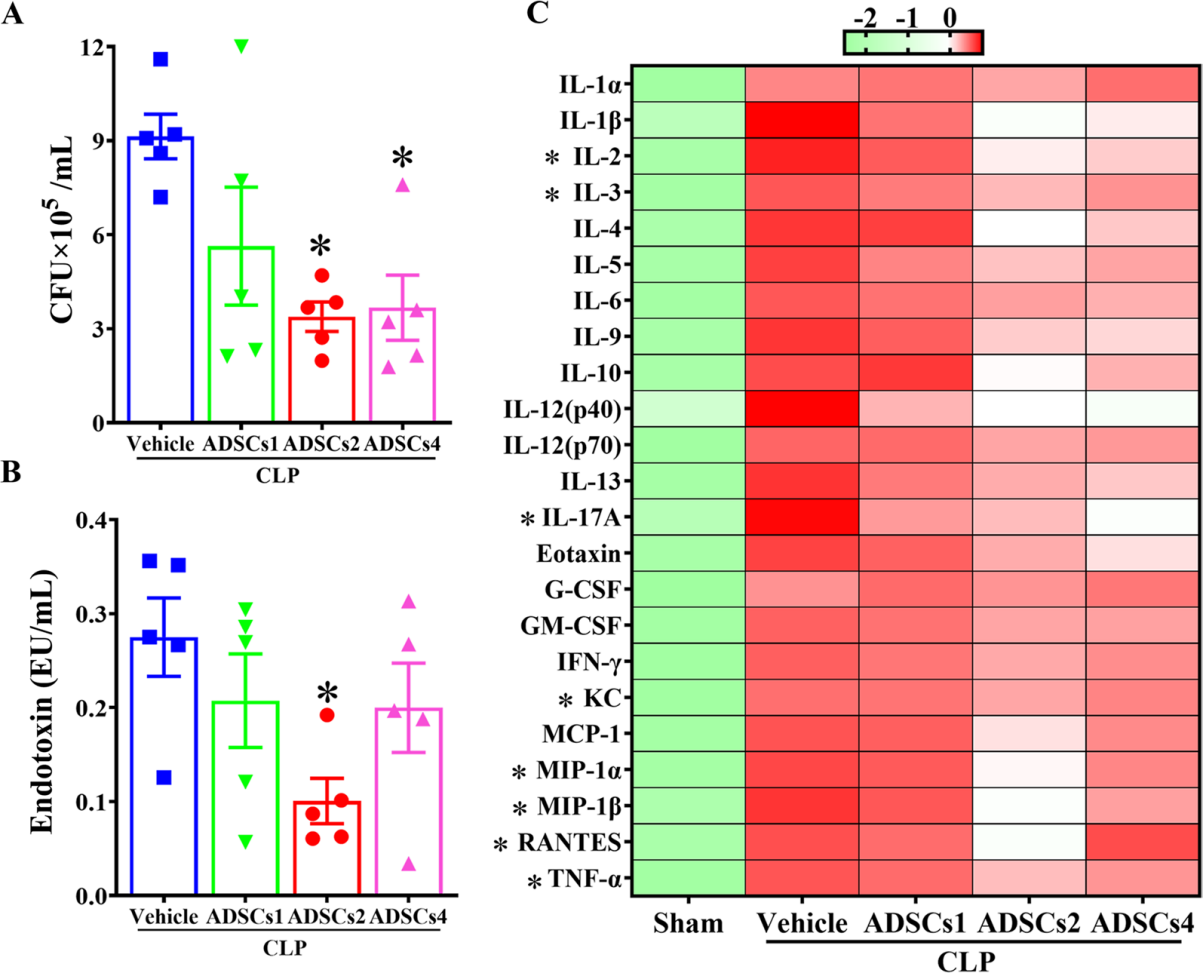
Effect of ADSCs treatment on CLP-induced bacterial burden and systemic inflammation. Blood samples were collected from CLP mice that received vehicle or different doses of ADSCs treatment at 6 h post-treatment. Whole blood samples were plated on Columbia blood agar plates with a 100-fold dilution in sterile saline and incubated overnight at 37 °C. Bacterial numbers were analyzed by colony forming units (CFU) (**A**). The plasma levels of endotoxin were measured by ELISA (**B**). Changes in systemic inflammation in CLP mice after ADSCs treatment were assessed by examining the plasma levels of 23 cytokines using a multiple cytokine assay. Plasma samples from sham-operated mice were used as reference controls. The expression patterns of cytokines were visualized by heatmap (**C**). *n* = 5 in each group. *, *p* < 0.05, significantly different from the CLP + Vehicle group

The measurement of blood bacterial burden showed that treatment with ADSCs2 or ADSCs4 significantly reduced the number of bacteria in the blood of CLP mice (Fig. 3A), but interestingly, only ADSCs2 treatment significantly reduced CLP-induced elevation of plasma endotoxin levels (Fig. 3B). A multiplex cytokine assay further demonstrated that ADSCs2 treatment effectively suppressed systemic inflammation in CLP mice, as evidenced by significant reductions in the plasma levels of pro-inflammatory cytokines, including IL-2, IL-3, KC, MIP-1*α*, MIP-1*β*, RANTES, and TNF-*α*. On the other hand, ADSCs4 treatment only significantly reduced plasma levels of IL-17, and ADSCs1 treatment did not cause any significant changes (Fig. 3C). Taken together, these data suggest that the therapeutic effect of intravenously infused ADSCs in septic mice is primarily attributable to the attenuation of bacterial burden and systemic inflammation.

### ADSCs treatment enhances phagocytic signaling and reduces cytokine production in PBMCs

**Fig. 4:**
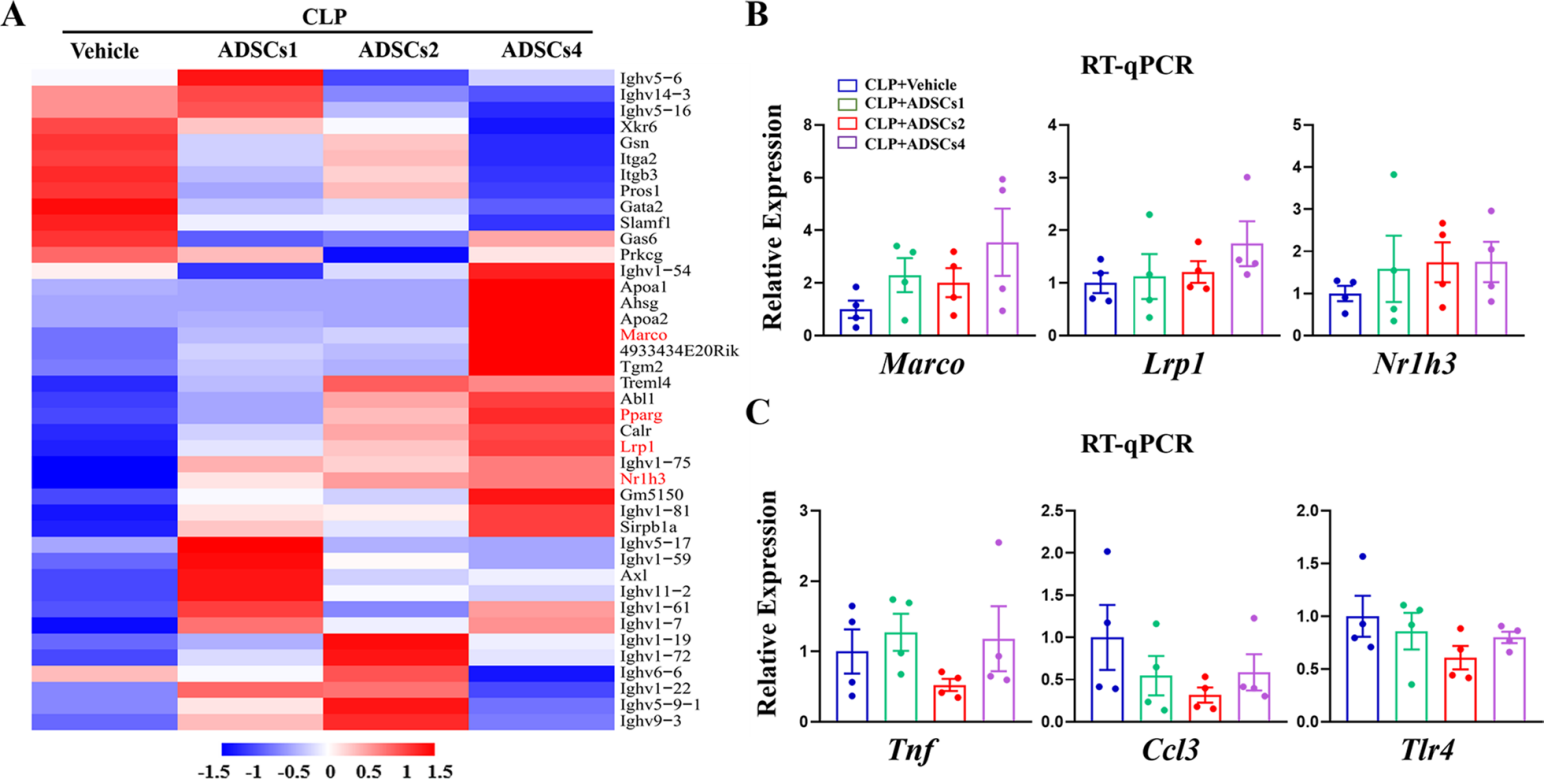
Effect of ADSCs treatment on phagocytic signaling and cytokine production in PBMCs. A total of 41 phagocytosis-related genes were significantly altered (|log2FC|> 1, FDR < 0.05) in PBMCs of CLP mice following ADSCs treatment, and visualized by heatmap with representative genes marked in red (**A**). RT-qPCR analysis was performed to validate the expression patterns of representative genes involved in phagocytosis (**B**) and inflammatory cytokine production (**C**) in PBMCs. *n* = 4 in each group

PBMCs play a pivotal role in the immune response against infection [26]; thus, we investigated whether the dose-specific antibacterial and immunomodulatory effects of ADSCs in CLP mice were related to the differential regulation of PBMCs function. Bulk RNA sequencing was performed to profile transcriptomic changes in PBMCs in different groups. Differentially expressed genes (DEGs) (|log2FC|> 1, FDR < 0.05) between different doses of ADSCs-treated and vehicle-treated groups were analyzed, and their numbers are listed in Additional file 9: Fig. S4A and B. Since intravenously infused MSCs exert their antibacterial properties in septic mice mainly by enhancing the phagocytic activity of blood monocytes [27], we first examined changes in phagocytic signaling in PBMCs after ADSCs treatment. Phagocytic signaling pathways, including Fc*γ*R-mediated phagocytosis (Additional file 10: Fig. S5A) and antigen processing and presentation (Additional file 10: Fig. S5B), were significantly activated in CLP mice treated with different doses of ADSCs compared to vehicle treatment. Furthermore, by intersecting genes involved in the phagocytosis process (GO: 0006909) with 1215 ADSCs-responsive genes in PBMCs (the collection of DEGs in three ADSCs-treated groups, see details in Additional file 4: Table S3), we identified 41 phagocytosis-related genes that were significantly altered in PBMCs following ADSCs treatment. Heatmap analysis showed that more than 70% (29/41) of these genes were significantly up-regulated after ADSCs treatment (Fig. 4A). Among them, the expression of phagocytic receptors (*Marco* and *Lrp1*) and transcription factors (*Nr1h3* and *Pparg*) involved in PBMCs-mediated bacterial clearance were significantly increased with raising doses of ADSCs infused in CLP mice. The expression trends of *Marco*, *Lrp1,* and *Nr1h3* were further validated by RT-qPCR analysis (Fig. 4B). Therefore, our data further demonstrated that the antibacterial effect of intravenously infused ADSCs in septic mice is mainly through enhancing the phagocytic capacity of PBMCs, possibly in a dose-dependent manner.

Next, to gain insight into the molecular mechanisms underlying the dose-specific changes in plasma levels of multiple cytokines in CLP mice after ADSCs treatment, we analyzed the expression patterns of cytokine genes in PBMCs. Heatmap analysis revealed a similar trend of reduction in the expression of the above-mentioned pro-inflammatory cytokines, including *Ccl3* (MIP-1*α*), *Ccl4* (MIP-1*β*), and *TNF* (TNF-*α*), in the ADSCs2-treated group (Additional file 10: Fig. S5C). Given the protective effect of ADSCs in reducing bacterial load and endotoxin levels in the blood of septic mice, the reduction in cytokine production in PBMCs may be due to attenuated endotoxin-mediated toll-like receptor signaling [28]. As expected, pathway analysis confirmed that toll-like receptor-mediated inflammatory cytokine production was significantly inhibited in the PBMCs of CLP mice after ADSCs2 treatment (Additional file 10: Fig. S5D). The expression patterns of several key representative genes, such as *Tnf, Ccl3*, and *Tlr4*, were then validated by RT-qPCR analysis (Fig. 4C).

Taken together, our findings demonstrate that intravenously infused ADSCs are dose-specific in modulating signaling pathways related to phagocytosis and cytokine production in PBMCs of septic mice, resulting in the aforementioned dose-specific antimicrobial and immunomodulatory effects.

### High-dose ADSCs treatment exacerbates coagulation dysfunction in septic mice

**Fig. 5:**
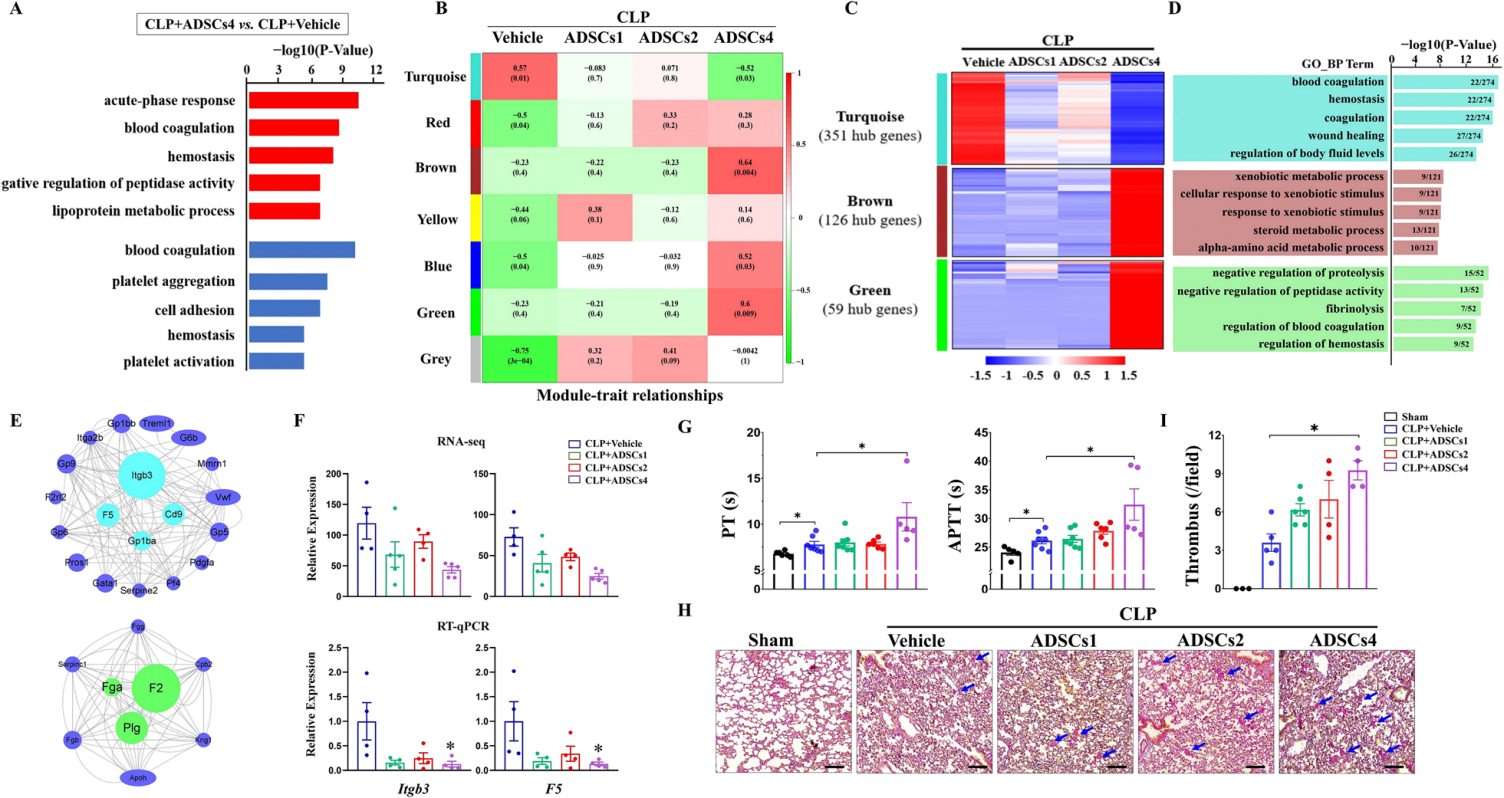
Effects of ADSCs treatment on CLP-induced coagulation dysfunction. GO analysis was performed to define biological processes enriched by DEGs between the CLP + ADSCs4 versus CLP + Vehicle groups (**A**). WGCNA was performed based on the 1215 ADSCs-responsive DEGs in PBMCs. Correlations between module eigengenes and different doses of ADSCs treatment were considered to be significant based on the criteria (correlation coefficients > 0.5 or < -0.5 and *p* value < 0.05) (**B**). The expression patterns of hub genes (|MM|> 0.8 and |GS|> 0.35) in the turquoise, brown and green modules were visualized by heatmap (**C**). GO analysis was performed to identify the annotated terms enriched for hub genes in each module, and the number of enriched genes was labeled on the corresponding GO term (**D**). Protein–protein interaction (PPI) network analysis was carried out to identify key driver (KD) genes responsible for the induction or inhibition of the coagulation pathway in PBMCs in the turquoise and green modules, respectively (**E**). Validation of the expression patterns of *Itgb3* and *F5* in PBMCs by RT-qPCR analysis. *n* = 4 in each group. *, *p* < 0.05, significantly different from the CLP + Vehicle group (**F**). Coagulation assays were performed to examine changes in plasma prothrombin time (PT) and activated partial thromboplastin time (APTT) at 6 h after vehicle or ADSCs treatment in CLP mice. Sham-operated mice served as controls. *n* = 5–7. *, *p* < 0.05 (**G**). Representative images demonstrating differences in fibrin deposition in the pulmonary vasculature in different treatment groups; fibrin deposition: blue arrow. Images were taken by Nikon 80i microscopy and SPOT Software at a resolution of 96 dpi, and processed in Adobe Photoshop at a resolution of 600 dpi. No downstream processing was utilized. Scar bar = 100 μm (**H**). Quantification of the number of thrombi observed per field in different groups. *n* = 3–6. *, *p* < 0.05 (**I**)

As described above, infusion of high-dose ADSCs in CLP mice resulted in unexpected acute death within 24 h after CLP surgery. To explore the potential causes, we first examined the transcriptomic changes in PBMCs after high-dose ADSCs treatment. Compared with the vehicle-treated group (CLP + Vehicle), a total of 971 (523 up- and 448 down-regulated) DEGs were detected in the high-dose ADSCs-treated group (CLP + ADSCs4) (Additional file 9: Fig. S4A and B). GO enrichment analysis revealed that both up- and down-regulated DEGs were mainly enriched in biological processes related to blood coagulation and hemostasis (Fig. 5A), suggesting that intravenous infusion of high-dose ADSCs may affect the coagulation system in septic mice.

To assess the correlation between ADSCs infusion dose and coagulation pathway gene expression in PBMCs, we performed weighted gene coexpression network analysis (WGCNA) using the 1215 ADSCs-responsive genes in PBMCs described above. As shown in Fig. 5B, four modules, designated turquoise, brown, blue, and green, showed strong correlations with ADSCs4 treatment, whereas no coexpression modules were found to be significantly associated with ADSCs1 or ADSCs2 treatments. Based on the cutoff criteria (|MM|> 0.8 and |GS|> 0.35), 351, 126, 135, and 59 highly connected hub genes corresponding to the turquoise, brown, blue, and green modules, respectively, were identified (Additional file 5: Table S4) and visualized by the heatmap shown in Fig. 5C. Interestingly, the expression of hub genes in the turquoise module exhibited a decreasing trend after ADSCs treatment, and these genes were mainly concentrated in processes related to blood coagulation and wound healing. In contrast, hub genes in the brown and green modules were significantly induced only by ADSCs4 treatment and were mainly enriched in metabolic processes (e.g., steroid and alpha-amino acid) and processes related to coagulation (e.g., fibrinolysis and blood coagulation) (Fig. 5D). No GO terms were enriched by hub genes in the blue module. Protein–protein interaction (PPI) network analysis identified *Itgb3*, *F5*, *Cd9*, and *Gp1ba* in the turquoise module and *F2*, *Plg* and *Fga* in the green module as key driver (KD) genes responsible for the induction or inhibition of the coagulation pathway in PBMCs following ADSCs4 treatment (Fig. 5E). The expression patterns of *Itgb3* and *F5* were then validated by RT-qPCR (Fig. 5F). Together, these data suggest that infusion of ADSCs in septic mice results in disturbance of coagulation signaling in PBMCs only at high doses.

To further investigate whether acute death in the CLP + ADSCs4 group was associated with aggravated coagulation dysfunction, we examined alterations in coagulation status. Coagulation assays showed that CLP mice had significantly prolonged prothrombin time (PT) and activated partial thromboplastin time (APTT) at 12 h after surgery compared to sham-operated mice. However, intravenous infusion of ADSCs in CLP mice at high doses resulted in further prolongation of PT and APTT (Fig. 5G). Consistently, MSB staining revealed that ADSCs4 treatment augmented fibrin deposition in the pulmonary vasculature of CLP mice compared with vehicle-treated groups (Fig. 5H, I).

Altogether, these data strongly imply that intravenous infusion of high-dose ADSCs exacerbates coagulation dysfunction and pulmonary thrombosis in septic mice, which may be responsible for the increased acute mortality in the high-dose ADSC-treated group.

### Dose-specific modulation of immune, metabolic and hypoxic responses in the livers of septic mice following ADSCs treatment

**Fig. 6:**
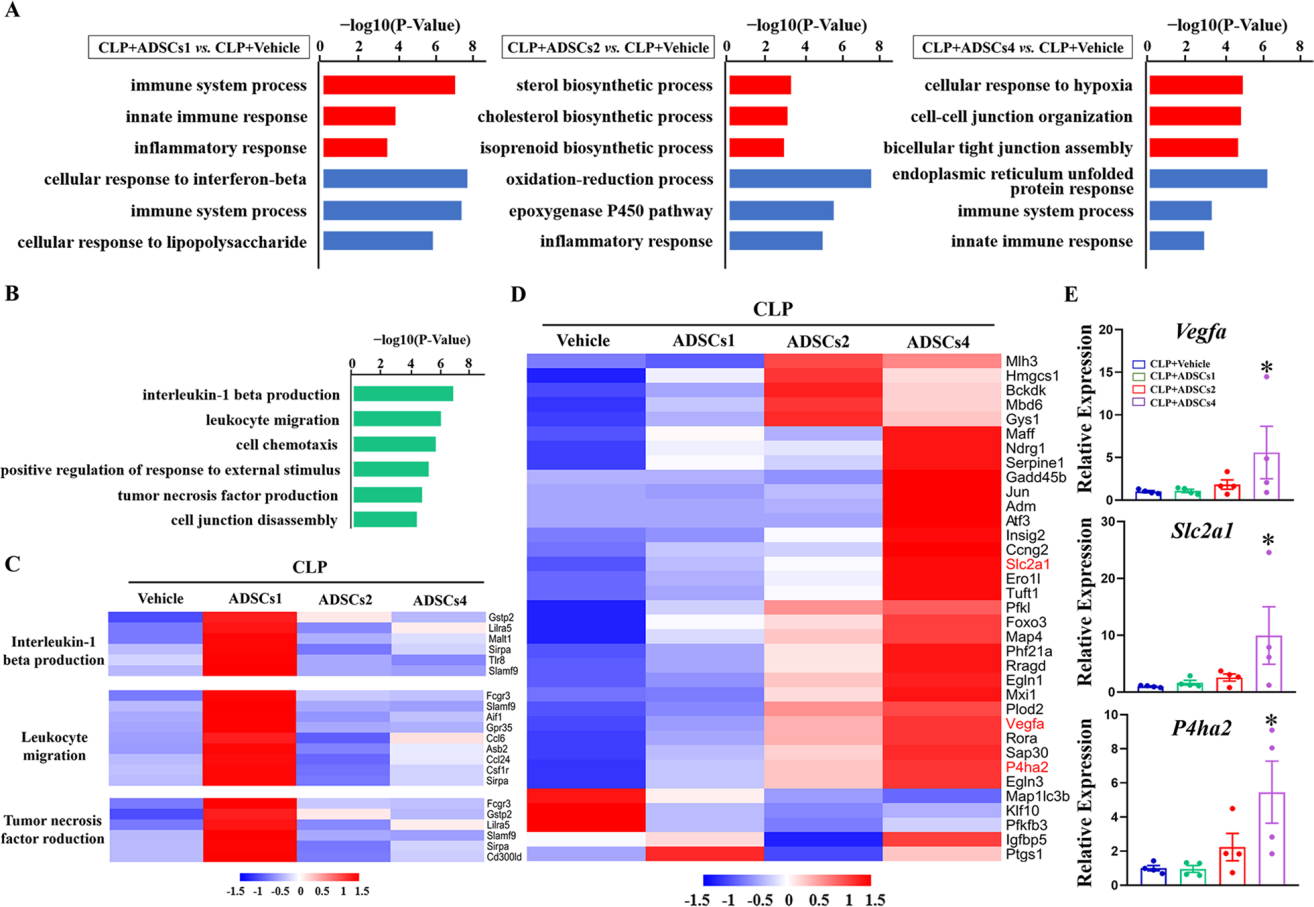
Dose-related effect of ADSCs on liver transcriptome changes in CLP mice. GO analysis was performed to define biological processes enriched by DEGs between different treatment groups (**A**) and the unique genes induced by ADSCs1 treatment (**B**). DEGs involved in interleukin-1 beta production, leukocyte migration and tumor necrosis factor production were visualized by heatmap (**C**). Heatmap visualization of the expression patterns of 35 hypoxia-inducible genes among ADSCs-responsive genes in the liver, with representative genes marked in red (**D**). Changes in the expression levels of *Vegfa*, *Slc2a1* and *P4ha2* in the liver were validated by RT-qPCR (**E**). *n* = 4 in each group. *, *p* < 0.05, significantly different from the CLP + Vehicle group

To further explore the dose-specific regulatory mechanisms by which ADSCs ameliorated acute multiple organ injury, especially in the liver, we performed hepatic transcriptome analysis at 6 h after vehicle or different doses of ADSCs treatment in CLP mice. Compared to the vehicle treatment, CLP mice treated with ADSCs1, ADSCs2, and ADSCs4 yielded 253 (184 up- and 69 down-regulated), 841 (501 up- and 340 down-regulated), and 967 (759 up- and 198 down-regulated) DEGs, respectively (Additional file 9: Fig. S4C and D). GO analysis revealed that down-regulated DEGs in all three ADSCs treatment groups were mainly enriched in processes related to inflammation and immune response, whereas up-regulated DEGs-enriched GO terms showed significant dose-specific differences. Specifically, up-regulated DEGs in the ADSCs1-treated group were unexpectedly concentrated in immune system processes and inflammatory responses, while genes up-regulated by ADSCs2 treatment were mainly associated with biosynthetic processes related to sterol, cholesterol and isoprenoid. Interestingly, ADSCs4 treatment activated the expression of genes involved in the cellular response to hypoxia and cell–cell junction organization (Fig. 6A).

To further understand the molecular basis of low-dose ADSCs-induced hepatic immune responses in CLP mice, we performed additional analysis on 90 unique DEGs (Additional file 9: Fig. S4D) that were specifically up-regulated in the CLP + ADSCs1 group. As shown in Fig. 6B, these unique ADSCs1-induced genes were mainly enriched in interleukin-1 beta production, leukocyte migration, and tumor necrosis factor production. The expression patterns of genes involved in these GO terms were visualized by heatmaps (Fig. 6C).

In addition, to gain insight into the changes in hypoxia signaling in the liver of CLP mice following high-dose ADSCs treatment, we applied the intersection between the previously validated hypoxia-inducible gene set [22] and 1522 ADSCs-responsive genes in the liver (the collection of DEGs in three ADSCs-treated groups, see details in Additional file 4: Table S3). As shown in Fig. 6D, 35 hypoxia-inducible genes were identified, most of which were highly up-regulated only after high-dose ADSC treatment. qPCR analysis further confirmed the expression patterns of several well-known hypoxia-responsive genes, such as *Vegfa*, *Slc2a1*, and *P4ha2* (Fig. 6E).

Collectively, these data indicate that ADSCs exert anti-inflammatory effects in the liver of septic mice regardless of the infusion dose; however, only moderate doses of ADSCs infusion can significantly restore hepatic metabolism, and lower or higher doses of ADSCs infusion may in turn augment the hepatic immune response and hypoxic liver injury, respectively.

## Discussion

In this study, we evaluated the dose–response effect of ADSCs therapy in a murine model of polymicrobial sepsis induced by CLP surgery. The three different IV doses of ADSCs were designed based on effective doses reported in previous preclinical studies, ranging from 1 × 10^7^ to 4 × 10^7^ cells/kg. We found that the optimal IV dose of ADSCs for sepsis treatment was 2 × 10^7^ cells/kg, whereas higher (4 × 10^7^ cells/kg) and lower (1 × 10^7^ cells/kg) doses were less or not effective. Consistent with previous studies [29–31], the therapeutic effect of ADSCs in sepsis demonstrated in this study is manifested by a significant improvement in survival and a reduction in sepsis-induced bacterial burden, endotoxemia, systemic inflammation, and multi-organ damage.

The mechanism of how intravenously infused MSCs exert their therapeutic effects in sepsis remains elusive. By tracking the distribution and retention of ADSCs in multiple organs of septic mice, we found that the majority of PKH26-labeled ADSCs accumulated in the lungs at 6 h after intravenous infusion, with only a smaller proportion of PKH26-positive signals appearing in the liver and spleen. This implies that the antibacterial, anti-inflammatory, and organ-protective effects of intravenously infused ADSCs demonstrated in this study are more likely related to indirect regulation of immune cell function rather than direct involvement in the repair of damaged tissue. This idea is further supported by the fact that signaling pathways related to phagocytosis and cytokine production were significantly enhanced and attenuated, respectively, in PBMCs of septic mice after ADSCs treatment. Furthermore, consistent with previous findings [32–35], we observed a gradual increase in the amount of PKH26-positive signals in the liver and spleen with a concomitant decrease in the lung after ADSCs infusion over time. However, whether PKH26-positive signals in the liver and spleen are viable ADSCs migrating from the pulmonary capillary bed or immune cells engulfing cellular debris of ADSCs, and their role in local tissue repair, remains to be further investigated.

Contradicting previous findings [30, 36–38], we did not observe any therapeutic effect of low-dose (1 × 10^7^ cells/kg) ADSCs in septic mice. One possible explanation for this conflicting result is that the murine model of sepsis employed in this study was induced by double-puncture CLP with an 18-gauge needle, which resulted in more severe sepsis than previous studies (up to 60% and 90% mortality at 24 h and 7 days, respectively). As a result, the antibacterial, immunomodulatory, and tissue-repair properties of ADSCs may not be sufficient at lower infusion doses to combat severe sepsis-induced bacteremia, systemic inflammation, and acute organ failure. Indeed, our transcriptomic data revealed that the expression of genes involved in bacterial phagocytosis, such as *Marco*, *Lrp1, Nr1h3*, and *Pparg*, in PBMCs of septic mice tended to increase with the infusion dose of ADSCs, suggesting that the ineffectiveness of low-dose ADSCs in sepsis is at least in part due to insufficient regulation of bacterial clearance mediated by PBMCs.

Moreover, by analyzing transcriptomic changes in the liver, we noticed that ADSCs may specifically exert both immunosuppressive and immunostimulatory effects at low infusion doses in septic mice, as indicated by the repression of genes involved in the cellular response to interferon-beta and the simultaneous activation of genes related to IL-1 production, leukocyte migration and TNF production. Therefore, the additional activation of the hepatic immune response by low-dose ADSCs therapy provides another explanation for their ineffectiveness in sepsis therapy. Further studies are needed to determine whether there is dose specificity in regulating the direction of MSCs-mediated immunomodulatory effects (i.e., immunoactivation or immunosuppression) in inflammatory disorders such as sepsis.

Surprisingly, in the present study, we found that ADSCs therapy at a high dose (4 × 10^7^ cells/kg) improved overall survival in septic mice but unexpectedly resulted in increased acute mortality. To explore the potential causes, we examined transcriptomic changes in PBMCs and the liver. Interestingly, we found that administration of high doses of ADSCs in septic mice specifically perturbed the expression of a series of genes involved in blood coagulation in PBMCs, such as *F2, Fgb, F5*, and *Itgb3*, suggesting that high-dose ADSCs infusion may affect the coagulation system in septic mice. Coagulation parameter examination further confirmed that intravenous infusion of ADSCs at high doses exacerbated CLP-induced coagulation dysfunction, as evidenced by further prolongation of PT and APTT in CLP mice. Akin to our results, a recent clinical study showed that intravenous infusion of allogeneic ADSCs elicited a transient procoagulant effect at high doses (4 × 10^6^ cells/kg, equal to 4 × 10^7^ cells/kg in mice) in LPS injection-induced human endotoxemia [39]. Nonetheless, no clinical adverse events, such as thrombosis, were reported in this clinical study, or in other MSCs trials using similar or even higher doses [40–42]. Likewise, we did not observe any acute response or death in sham-operated mice treated with high-dose ADSCs, indicating that the procoagulant activity of ADSCs in sepsis may also depend on the host coagulant status.

Considering that the severe sepsis model used in the present study results in more severe coagulation disorders [43, 44], the additional procoagulant effect of high-dose ADSCs may greatly increase the risk of thrombosis and consequently lead to acute mortality in septic mice. This idea is supported by the finding that fibrin deposition in the pulmonary vasculature was significantly augmented in CLP mice that received high-dose ADSCs treatment. Furthermore, we noticed that a number of genes involved in the hypoxia response were activated in the liver of septic mice after high-dose ADSCs treatment, which may be a manifestation of hepatic hypoxic injury due to enhanced intrahepatic microthrombosis [45]. Curiously, high-dose MSCs infusion-induced acute mortality in septic mice has not been described in previous studies [46, 47]. This contradiction can be explained by the fact that ADSCs exhibited higher surface tissue factor (TF) expression levels than the source of MSCs (i.e., umbilical cord blood and bone marrow) used in other studies [48–50], which makes them more likely to elicit a procoagulant response after systemic intravenous infusion [39, 48]. Therefore, we propose that the acute death caused by high-dose ADSCs treatment in this study is related to the exacerbation of coagulation dysfunction and thrombosis in septic mice. To provide more direct evidence for this hypothesis, future studies will focus on combining high-dose ADSCs with anticoagulation therapy to assess whether it can reduce the incidence of acute death and further improve the efficacy of ADSCs in sepsis.

Interestingly, a recent study conducted a comprehensive analysis of 914 MSCs trials from ClinicalTrials.gov and indicated that there may be minimal effective doses (MEDs) of MSCs for the treatment of human diseases, ranging from 100 to 150 million cells/patient (equaling 1.4 × 10^6^ to 2.1 × 10^6^ cells/kg in a 70 kg human), with lower or higher doses being less effective [7]. Coincidentally, the optimal IV dose for ADSCs identified in the present study is roughly equivalent to 2 × 10^6^ cells/kg in humans scaled by body surface area, which fits well within the suggested MED range. Moreover, a recent dose–response study in a rat model of fecal sepsis also demonstrated that intravenous infusion of human umbilical cord-derived MSCs (hUC-MSCs) at a dose of 1 × 10^7^ cells/kg (roughly equivalent to 2 × 10^6^ cells/kg in humans) exhibited the greatest improvement in reducing bacterial burden, peritoneal leukocyte infiltration and mortality [51]. However, the single infusion dose of MSCs most commonly used in current clinical trials of sepsis treatment is 1 × 10^6^ cells/kg [8–10], which is lower than the optimal therapeutic dose described above. Thus, the low efficacy of MSCs therapy in septic patients reported in previous phase I/II studies is more likely to be attributable to inappropriate infusion doses of MSCs rather than a lack of statistical power limited by the small sample size [8–11].

An obvious limitation of this study is that unlike the clinical standard of care for septic patients [3], we performed only fluid resuscitation (saline, s.c.) but no antibiotics after CLP surgery. Nonetheless, several recent meta-analyses of previous preclinical studies suggest that additional fluid resuscitation or antibiotic treatment did not affect the protective role of MSCs in sepsis [5, 6]. However, considering the beneficial role of antibiotics in alleviating sepsis-induced bacterial burden and cytokine storms [52], the lack of antibiotic therapy in the present study may contribute to an overall decrease in the therapeutic efficacy of ADSCs. Therefore, future studies need to employ more clinically relevant therapeutic interventions, such as fluid resuscitation in combination with antibiotic treatment, to obtain precise optimal dose ranges of ADSCs for the treatment of sepsis.

## Conclusions

In this study, we demonstrate that the therapeutic efficacy of ADSCs in sepsis is highly dependent on their infusion dose. The ideal IV dose of ADSCs is approximately 2 × 10^7^ cells/kg body weight in mice, with lower and higher doses being less effective. The dose-specific therapeutic effect of ADSCs on sepsis revealed in this study will provide an important reference for the optimization of MSCs dosing regimens in future sepsis clinical trials.

## Supplementary Information

**Additional file 1: Fig. S1. **Characterization of the CLP-induced murine model of sepsis. Mice were subjected to sham or CLP surgery, and the core body temperature (**A**) and murine sepsis score (**B**) were monitored at 6 h after CLP surgery. Mice were subsequently killed to obtain blood and tissue samples. Plasma levels of endotoxin (**C**) and inflammatory cytokines (**D**), including IL-1*β*, IL-6, IL-10 and TNF-*α*, were determined by ELISA. Plasma levels of biochemical indicators, including ALT, AST, BUN and LDH, were determined by autoanalyzer (**E**). Histological examination was performed in the lungs (alveolar septal inflammation: blue arrow; alveolar collapse: dotted line), livers (abnormal hyperplasia of bile duct epithelium: blue arrow; hepatocyte disarrangement and hydropic degeneration: dotted line) and kidneys (acute tubular necrosis: red arrow; glomerular damage: black arrow; renal interstitial infiltration: blue arrow) of septic mice using hematoxylin and eosin (H&E) staining. Images were taken by Nikon 80i microscopy and SPOT Software at a resolution of 96 dpi, and processed in Adobe Photoshop at a resolution of 600 dpi. No downstream processing was utilized. Scar bar=200 μm. Organ injury was analyzed by double-blind pathology scoring (**F**). Survival after surgery was assessed every 12 h for 168 hours. n=3–10 (**G**). *, *p*<0.05, significantly different from the sham group.

**Additional file 2: Table S1**. Raw read counts of genes for RNA-Seq in PBMCs and liver.

**Additional file 3: Table S2.** Gene Ontology (GO) enrichment analysis of DEGs between different groups in PBMCs and liver.

**Additional file 4: Table S3.** List of ADSCs-responsive genes in PBMCs and liver.

**Additional file 5: Table S4. **List of hub genes in correlated modules in PBMCs.

**Additional file 6: Table S5**. List of genes involved in the phagocytosis and hypoxia responses.

**Additional file 7: Fig. S2. **Characterization of mouse ADSCs. Representative morphology of mouse ADSCs was observed by Olympus CKX53 microscopy. Images were taken by ImageView software at a resolution of 96 dpi. Scar bar=500 μm (**A**). Cellular surface markers of ADSCs, including positive markers (CD29 and CD44) and negative markers (CD31 and CD45), were analyzed by flow cytometry (**B**). ADSCs were induced to differentiate into adipocytes (left), osteocytes (middle), and chondrocytes (right). Images of adipogenic and osteogenic differentiation were acquired by Olympus CKX53 microscopy and ImageView software at a resolution of 96 dpi. Images of chondrogenic differentiation were acquired by Nikon 80i microscopy and SPOT Software at a resolution of 96 dpi. Scar bar=200 μm (**C**). Immunofluorescence staining of Oct4 (green) in ADSCs. The nucleus was stained with DAPI (blue). Images were acquired using a Nikon A1 laser confocal microscope and NIS-Elements software at a resolution of 96 dpi. Scale bar=100 μm (**D**). All images were processed in Adobe Photoshop at a resolution of 600 dpi. No downstream processing was utilized.

**Additional file 8: Fig. S3. **Dynamic distribution of intravenously infused ADSCs in multiple organs of CLP mice. PKH26-labeled positive signals (red) were detected in the lung (**A**), liver (**B**), spleen (**C**), heart (**D**), and kidney (**E**) by fluorescence imaging at 12, 24, and 72 h after CLP using a Nikon A1 laser confocal microscope. Images were taken by NIS-Elements software at a resolution of 1200 dpi. No downstream processing was utilized. The nucleus was stained with DAPI (blue). CLP mice treated with vehicle solution served as the negative control. *n*=3 in each group. Scale bar=500 µm.

**Additional file 9: Fig. S4.** The number of DEGs between different groups in PBMCs and liver. Bulk RNA sequencing was performed to profile transcriptomic changes in PBMCs and liver in different groups. The number of differentially expressed genes (DEGs) (|log2FC|>1, FDR<0.05) in PBMCs (**A**) and liver (**C**) were listed. Venn diagrams were employed to show the number of unique and overlapping DEGs between different groups in PBMCs (**B**) and liver (**D**).

**Additional file 10: Fig. S5.** Changes in phagocytic and cytokine production signaling pathways in PBMCs after ADSCs treatment in CLP mice. The expression patterns of genes involved in Fc*γ*R-mediated phagocytosis (**A**) and antigen processing and presentation (**B**) were analyzed and visualized using the Pathview web server. The expression of pro-inflammatory cytokines in PBMCs was visualized by heatmap (**C**). The status of the Toll-like receptor signaling pathway was analyzed and visualized using the Pathview web server (**D**). Left: CLP+ADSCs1 vs. CLP+Vehicle; Middle: CLP+ADSCs2 vs. CLP+Vehicle; Right: CLP+ADSCs4 vs. CLP+Vehicle. Permission has been obtained from Kanehisa laboratories for using KEGG pathway images [53].

## Data Availability

All data generated and/or analyzed during this study are available from the corresponding author upon reasonable request. The RNA-seq data reported in this article has been deposited in NCBI’s Gene Expression Omnibus (GEO) and are accessible through GEO Series accession number GSE214746.

## Abbreviations

ADSCs: Adipose-derived mesenchymal stem cells
ALT: Alanine transaminase
APTT: Activated partial thromboplastin time
AST: Aspartate aminotransferase
BP: Biological process
BUN: Blood urea nitrogen
*Cd9*: Cluster-of-differentiation antigen 9
CLP: Cecal ligation and puncture
DAPI: 4′,6-Diamidino-2-phenylindole
DEGs: Differentially expressed genes
*Egln1*: Egl-9 family hypoxia-inducible factor 1
ELISA: Enzyme-linked immunosorbent assay
EU: Endotoxin unit
*F2*: Coagulation factor II
*F5*: Coagulation factor V
FBS: Fetal bovine serum
FC: Fold change
FDR: False discovery rate
GO: Gene ontology
*Fga*: Fibrinogen alpha chain
*Gp1ba*: Glycoprotein Ib-alpha
H&E: Hematoxylin and eosin
hUC-MSCs: Human umbilical cord-derived MSCs
IL: Interleukin
IV: Intravenous
*Itgb3*: Integrin subunit beta 3
KC: Keratinocyte-derived chemokine
KD: Key driver
LDH: Lactate dehydrogenase
*Lrp1*: LDL receptor-related protein 1
*Macro*: Macrophage receptor with collagenous structure
MED: Minimum effective dose
MIP: Macrophage inflammatory protein
MSB: Martius Scarlet Blue
MSCs: Mesenchymal stem cells
MSS: Murine sepsis score
*Nr1h3*: Nuclear receptor subfamily 1 group H member 3
Oct4: Octamer-binding transcription factor 4
*P4ha2*: Prolyl 4-hydroxylase subunit alpha 2
PBS: Phosphate-buffered saline
*Plg*: Plasminogen
PMBCs: Peripheral blood mononuclear cells
*Pparg*: Peroxisome proliferator-activated receptor gamma
PPI: Protein–protein interaction
PT: Prothrombin time
RANTES: Regulated upon activation, normal T-cell expressed and presumably secreted
RIMS: Refractive index matching solution
RIN: RNA integrity number
RNA-seq: RNA sequencing
RT-qPCR: Real-time quantitative polymerase chain reaction
SEM: Standard error of mean
*Slc2a1*: Solute carrier family 2 member 1
TATc: Thrombin–antithrombin complexes
*TBP*: TATA-box-binding protein
TF: Tissue factor
*Tlr4*: Toll-like receptor 4
TNF: Tumor necrosis factor
*Vegfa*: Vascular endothelial growth factor A
WGCNA: Weighted correlation network analysis
*α*-MEM: *α*-Modified Eagle’s medium
