# Supplementary figures and images for: Dose-specific efficacy of adipose-derived mesenchymal stem cells in septic mice
Source: Stem Cell Res Ther. 2023 Feb 19;14:32. doi: 10.1186/s13287-023-03253-3 (PMC9940377; doi:10.1186/s13287-023-03253-3)

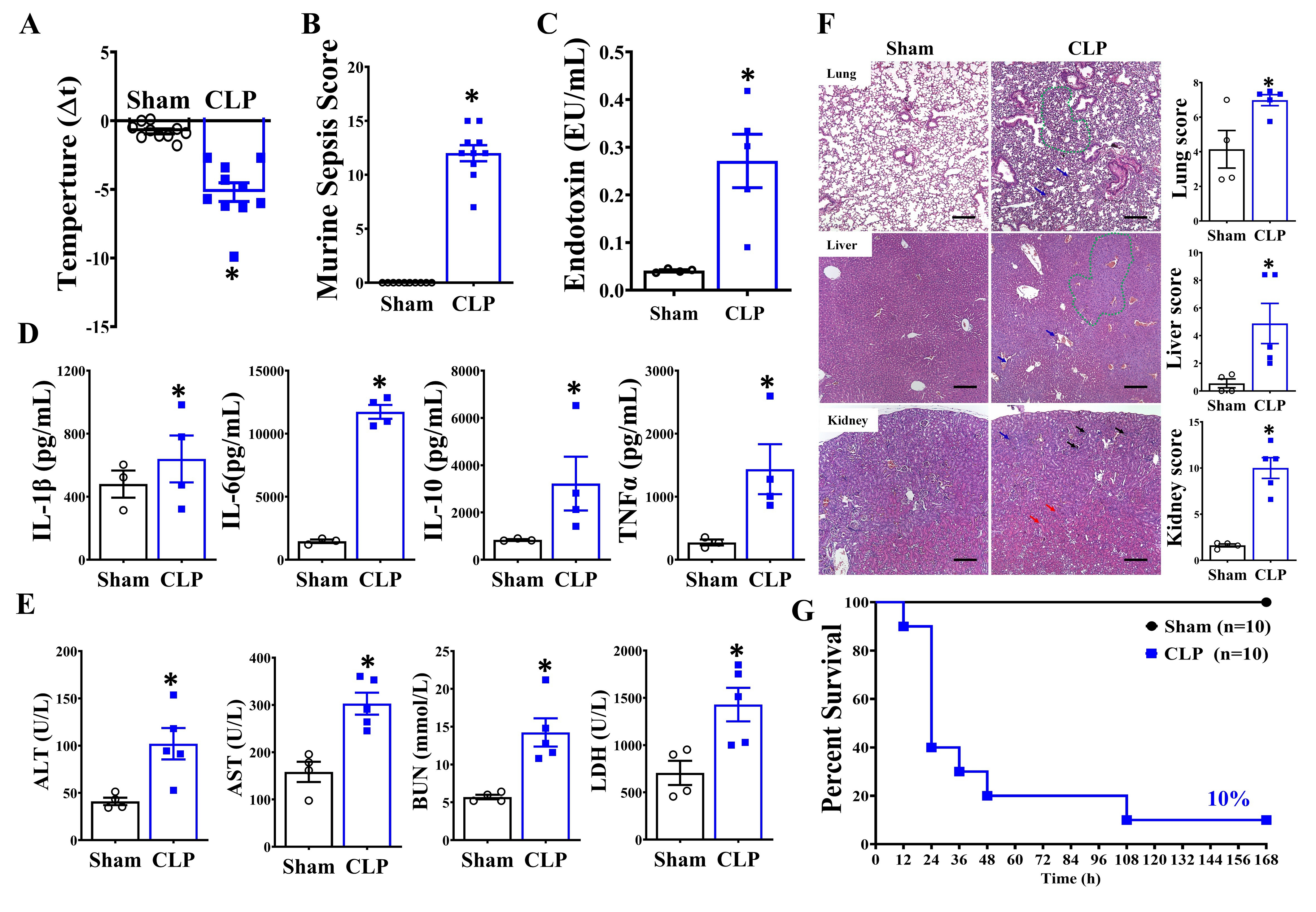

Supplement: Supplementary file 1 — Additional file 1: Fig. S1. Characterization of the CLP-induced murine model of sepsis. Mice were subjected to sham or CLP surgery, and the core body temperature (A) and murine sepsis score (B) were monitored at 6 h after CLP surgery. Mice were subsequently killed to obtain blood and tissue samples. Plasma levels of endotoxin (C) and inflammatory cytokines (D), including IL-1β, IL-6, IL-10 and TNF-α, were determined by ELISA. Plasma levels of biochemical indicators, including ALT, AST, BUN and LDH, were determined by autoanalyzer (E). Histological examination was performed in the lungs (alveolar septal inflammation: blue arrow; alveolar collapse: dotted line), livers (abnormal hyperplasia of bile duct epithelium: blue arrow; hepatocyte disarrangement and hydropic degeneration: dotted line) and kidneys (acute tubular necrosis: red arrow; glomerular damage: black arrow; renal interstitial infiltration: blue arrow) of septic mice using hematoxylin and eosin (H&E) staining. Images were taken by Nikon 80i microscopy and SPOT Software at a resolution of 96 dpi, and processed in Adobe Photoshop at a resolution of 600 dpi. No downstream processing was utilized. Scar bar=200 μm. Organ injury was analyzed by double-blind pathology scoring (F). Survival after surgery was assessed every 12 h for 168 hours. n=3–10 (G). *, p<0.05, significantly different from the sham group. [file 13287_2023_3253_MOESM1_ESM.tif]

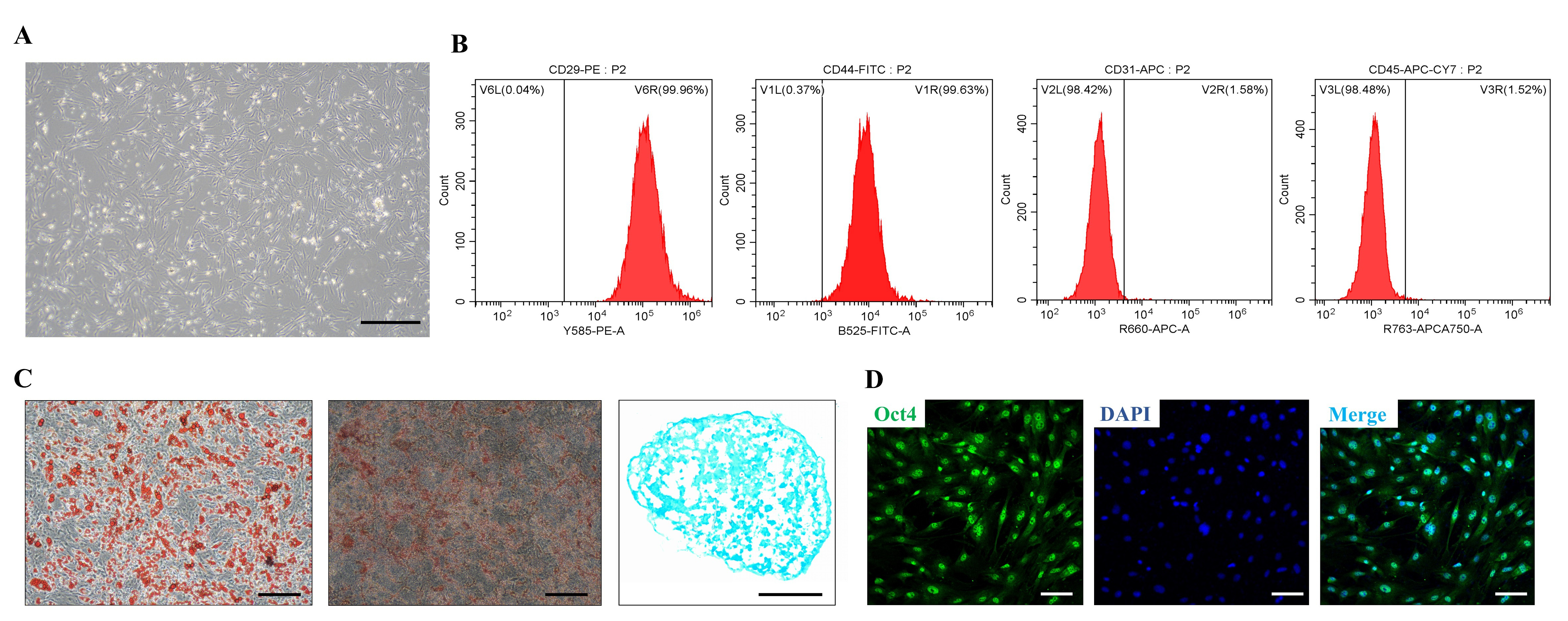

Supplement: Supplementary file 7 — Additional file 7: Fig. S2. Characterization of mouse ADSCs. Representative morphology of mouse ADSCs was observed by Olympus CKX53 microscopy. Images were taken by ImageView software at a resolution of 96 dpi. Scar bar=500 μm (A). Cellular surface markers of ADSCs, including positive markers (CD29 and CD44) and negative markers (CD31 and CD45), were analyzed by flow cytometry (B). ADSCs were induced to differentiate into adipocytes (left), osteocytes (middle), and chondrocytes (right). Images of adipogenic and osteogenic differentiation were acquired by Olympus CKX53 microscopy and ImageView software at a resolution of 96 dpi. Images of chondrogenic differentiation were acquired by Nikon 80i microscopy and SPOT Software at a resolution of 96 dpi. Scar bar=200 μm (C). Immunofluorescence staining of Oct4 (green) in ADSCs. The nucleus was stained with DAPI (blue). Images were acquired using a Nikon A1 laser confocal microscope and NIS-Elements software at a resolution of 96 dpi. Scale bar=100 μm (D). All images were processed in Adobe Photoshop at a resolution of 600 dpi. No downstream processing was utilized. [file 13287_2023_3253_MOESM7_ESM.tif]

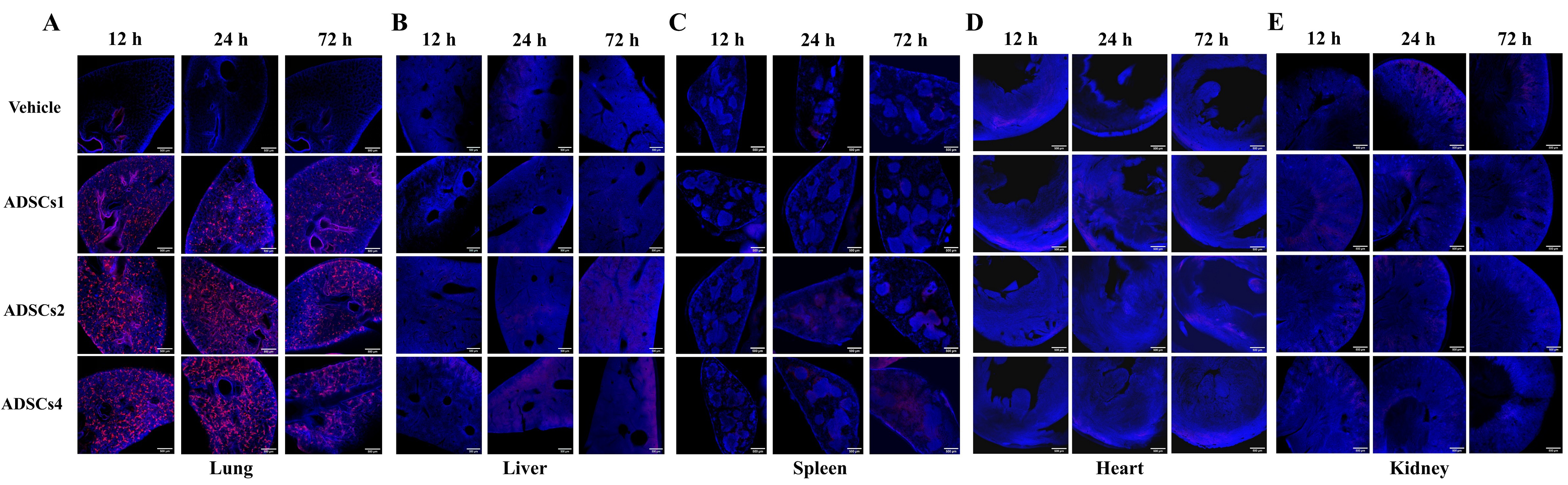

Supplement: Supplementary file 8 — Additional file 8: Fig. S3. Dynamic distribution of intravenously infused ADSCs in multiple organs of CLP mice. PKH26-labeled positive signals (red) were detected in the lung (A), liver (B), spleen (C), heart (D), and kidney (E) by fluorescence imaging at 12, 24, and 72 h after CLP using a Nikon A1 laser confocal microscope. Images were taken by NIS-Elements software at a resolution of 1200 dpi. No downstream processing was utilized. The nucleus was stained with DAPI (blue). CLP mice treated with vehicle solution served as the negative control. n=3 in each group. Scale bar=500 µm. [file 13287_2023_3253_MOESM8_ESM.tif]

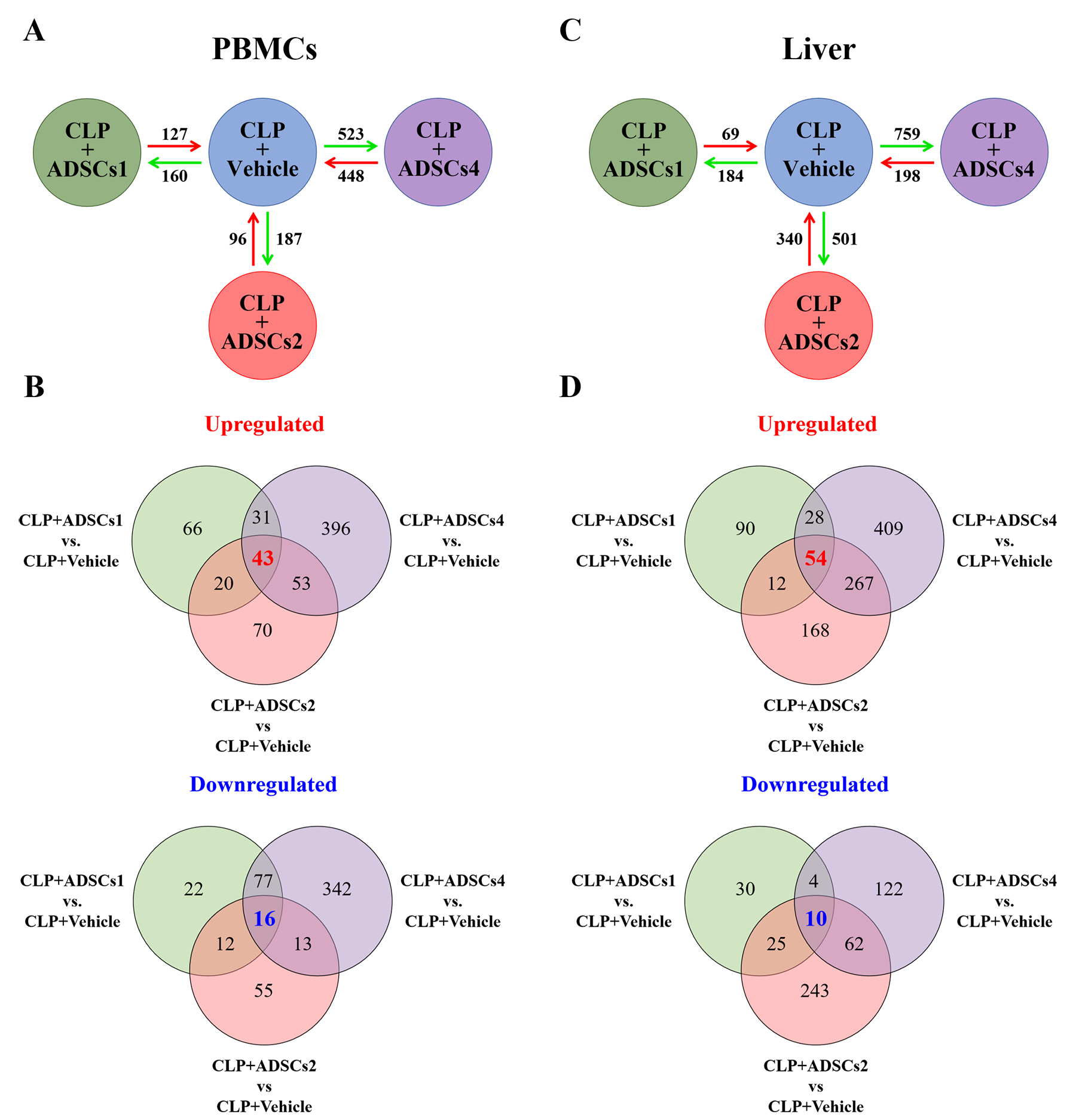

Supplement: Supplementary file 9 — Additional file 9: Fig. S4. The number of DEGs between different groups in PBMCs and liver. Bulk RNA sequencing was performed to profile transcriptomic changes in PBMCs and liver in different groups. The number of differentially expressed genes (DEGs) (|log2FC|>1, FDR<0.05) in PBMCs (A) and liver (C) were listed. Venn diagrams were employed to show the number of unique and overlapping DEGs between different groups in PBMCs (B) and liver (D). [file 13287_2023_3253_MOESM9_ESM.tif]

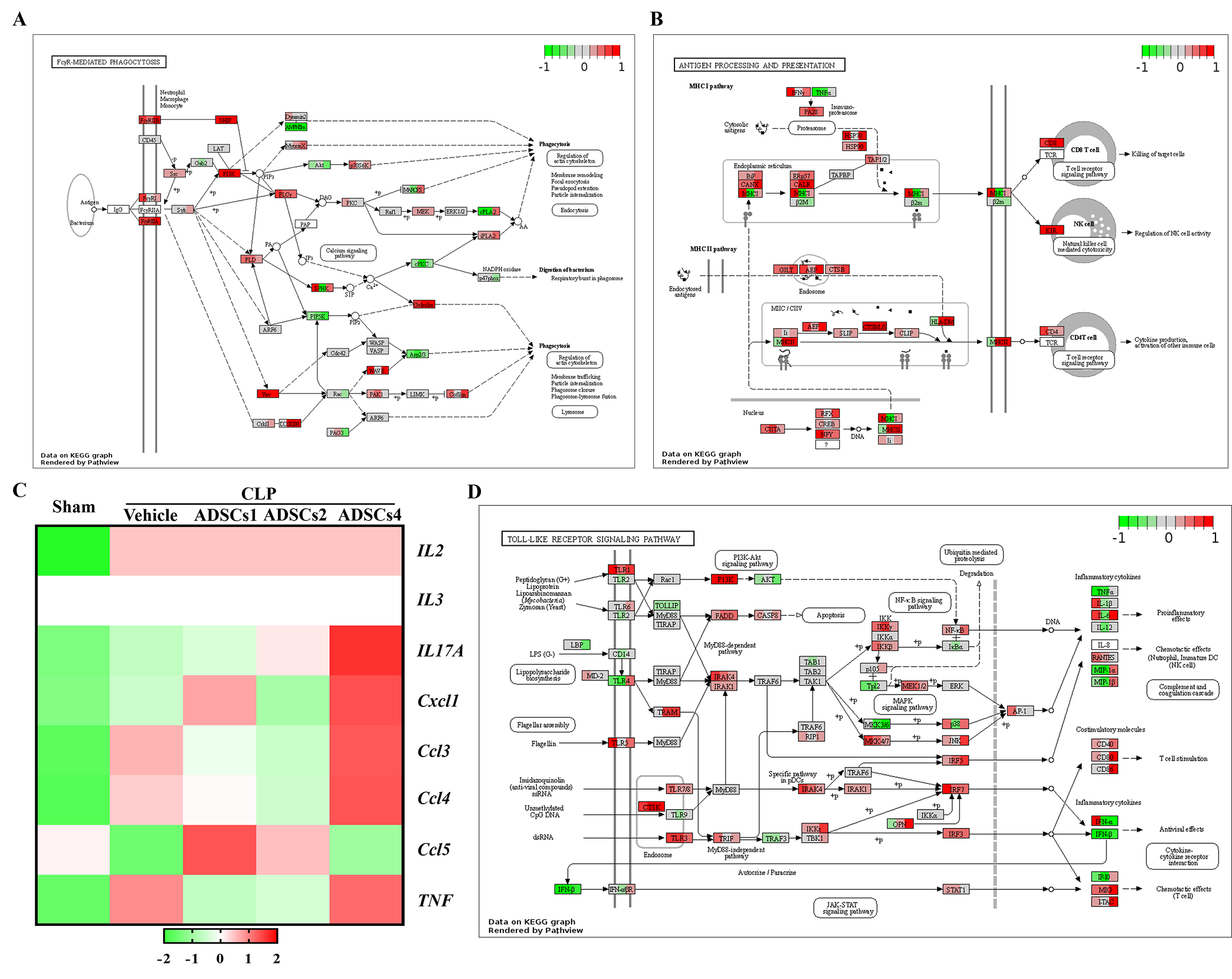

Supplement: Supplementary file 10 — Additional file 10: Fig. S5. Changes in phagocytic and cytokine production signaling pathways in PBMCs after ADSCs treatment in CLP mice. The expression patterns of genes involved in FcγR-mediated phagocytosis (A) and antigen processing and presentation (B) were analyzed and visualized using the Pathview web server. The expression of pro-inflammatory cytokines in PBMCs was visualized by heatmap (C). The status of the Toll-like receptor signaling pathway was analyzed and visualized using the Pathview web server (D). Left: CLP+ADSCs1 vs. CLP+Vehicle; Middle: CLP+ADSCs2 vs. CLP+Vehicle; Right: CLP+ADSCs4 vs. CLP+Vehicle. Permission has been obtained from Kanehisa laboratories for using KEGG pathway images [53]. [file 13287_2023_3253_MOESM10_ESM.tif]
